# Multiple samples aCGH analysis for rare CNVs detection

**DOI:** 10.1186/2043-9113-3-12

**Published:** 2013-06-11

**Authors:** Maciej Sykulski, Tomasz Gambin, Magdalena Bartnik, Katarzyna Derwińska, Barbara Wiśniowiecka-Kowalnik, Paweł Stankiewicz, Anna Gambin

**Affiliations:** 1 Institute of Informatics, University of Warsaw, Warsaw, Poland; 2 Institute of Computer Science, Warsaw University of Technology, Warsaw, Poland; 3 Department of Medical Genetics, Institute of Mother and Child, Warsaw, Poland; 4 Department of Molecular and Human Genetics, Baylor College of Medicine, Houston, TX, USA; 5 Mossakowski Medical Research Centre, Polish Academy of Sciences, Warsaw, Poland

## Abstract

**Background:**

DNA copy number variations (CNV) constitute an important source of genetic variability. The standard method used for CNV detection is array comparative genomic hybridization (aCGH).

**Results:**

We propose a novel multiple sample aCGH analysis methodology aiming in rare CNVs detection. In contrast to the majority of previous approaches, which deal with cancer datasets, we focus on constitutional genomic abnormalities identified in a diverse spectrum of diseases in human. Our method is tested on exon targeted aCGH array of 366 patients affected with developmental delay/intellectual disability, epilepsy, or autism. The proposed algorithms can be applied as a post–processing filtering to any given segmentation method.

**Conclusions:**

Thanks to the additional information obtained from multiple samples, we could efficiently detect significant segments corresponding to rare CNVs responsible for pathogenic changes. The robust statistical framework applied in our method enables to eliminate the influence of widespread technical artifact termed ‘waves’.

## Background

DNA Copy–Number Variations (CNVs), gains (duplications, triplications, amplifications) or losses (deletions) of chromosomal material, are known to underlie many types of constitutional genomic disorders and many cancer types [1,2].

Microarray–based genomic copy–number analysis became a standard practice in patients with diagnoses, including developmental delay (DD)/intellectual disability (ID), autism, and congenital anomalies. Array comparative genomic hybridization (aCGH) is now widely used for identification of segmental copy–number alterations in disease genomes [3]. In a typical experiment, DNA is extracted from two genomic samples (test vs reference) and labeled differently. Samples are mixed together and then hybridized to a microarray spotted with DNA probes. Signal fluorescent intensities of each spot from both samples are considered to be proportional to the amount of respective genomic sequence present.

Assigning significance to signals found in aCGH data is a challenging task, involving statistical analysis, and human verification by geneticists. It’s an ongoing effort: improving, automating, and verifying protocols for detection of rare CNVs which underlie diverse spectrum of diseases in human [4-6].

### Motivation and related research

In recent years there has been an increase in number of probes on the array in aCGH technology – the genomic resolution has improved. Designed high resolution arrays target in detection of changes in single exons, small as several hundred base pairs in size, and facilitate a better detection of CNVs. This helps in clinical interpretation of changes in patients with various clinical phenotypes, especially when a CNV overlaps with a gene known to be causative of the observed clinical phenotype [5].

The progress in array resolution increases challenges in aCGH data analysis. The main goal when analyzing aCGH data is to identify genomic regions with rearrangements. The specific challenge in clinical genetic diagnostics is to detect strictly pathogenic CNVs [7].

The primary hallmark of CNV’s pathogenicity is its rarity in the population. CNV is considered rare if it is not polymorphic. An aCGH sample signifies a rare CNV if it differs significantly from other samples in the same genomic region.

**Figure 1:**
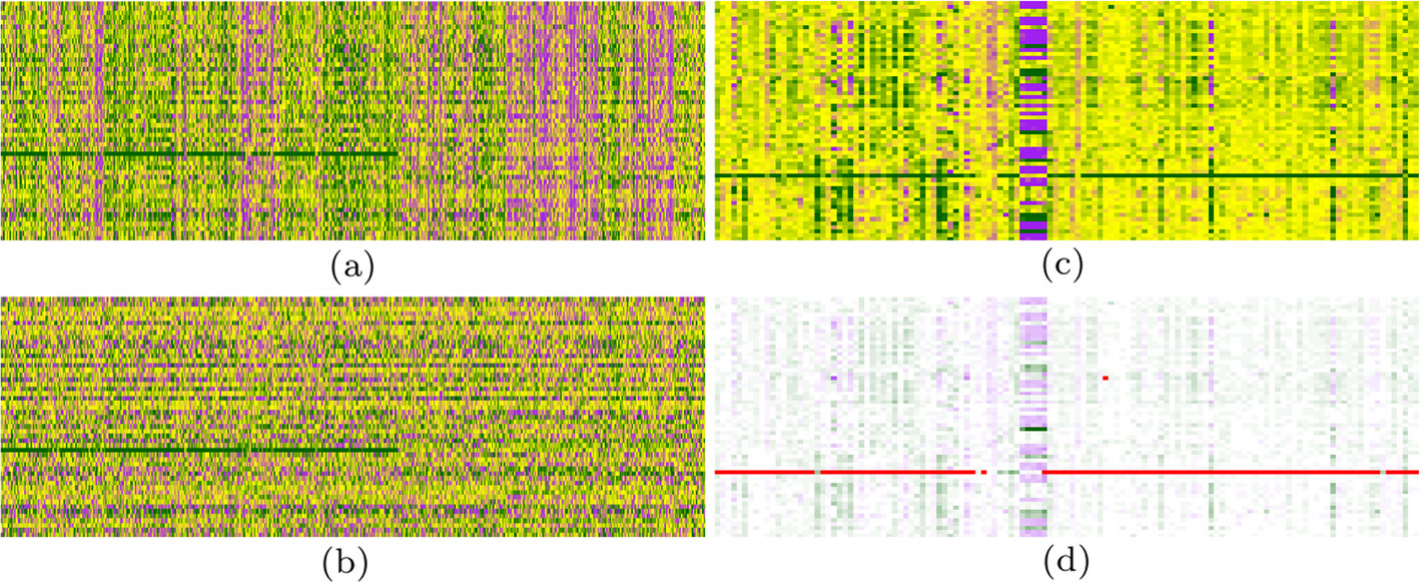
**Processing of logratio data.** In each subfigure, rows corresponds to samples and columns to probes. On the left: the effect of rank transformation; the same fragment of the genome represented by logratios **(a)** and their column ranks **(b)**. The wave pattern is eliminated, while true signal (clear deletion) is strengthen. On the right: the polymorphic region in the middle is surrounded by wave patterns and only one significant deletion is visible **(c)**; markers found by our algorithm indicate only deleted segment, all other spurious signals are ignored **(d)**.

Detection of CNVs from aCGH data is a process of separation from noise contiguous blocks of signal along a patient’s genome (Figure 1). This analysis process is called segmentation, and there are plethora of methods and algorithms for detection of CNVs through segmentation.

Segmentation methods to identify and describe the structure of the intervals use a variety of approaches, such as Gaussian models [8], hidden Markov models [9], wavelets [10] and quantile regression [11].

However, all the mentioned methods have two significant drawbacks: computational complexity and single sample restriction. The computational efficiency is a crucial parameter when one intends to analyze samples from high–resolution arrays (more than 100 K probes). Unfortunately, most of the sophisticated statistical methods for analyzing aCGH data have been tested on simulated artificial datasets or on Snijders dataset [12]. This dataset consists of 15 human cell lines DNA samples hybridized onto a CGH-array of 2276 probes. Clearly, the size of the typical clinical data is at least of two orders of magnitude larger.

Several methods for the simultaneous analysis of many aCGH samples have been published recently [13-15]. They turned out to be very useful for analyzing cancer data because one can exploit frequent rearrangement patterns.

However, when studying genomic disorders one has to eliminate non–pathogenic polymorphisms and a quite different approach should be applied, see e.g. SCOUT method for rare CNVs developed for SNP microarrays [16].

Another obstacle, that may have an adverse effect on aberration calling is the presence of waves. Waviness, hypothesized to be correlated with the GC content or replication timing of the probes was first observed for high resolution CGH arrays, and recently detected in other platforms based on DNA hybridization, e.g. ChIP-on-chip DNA methylation studies [17,18]. Whereas the presence of waves is clear, the mechanism underlying them remains unexplained. Many segmentation methods make use of the apparent breakpoint structures and detect too many segments when the waves are present. Therefore, segmentation result is significantly biased (the phenomenon occurs especially in tumor samples [19]) and requires both wave smoothing preprocessing and rigorous experimental validation (e.g. using Fluorescence in situ hybridization (FISH)).

Thus, the set of rearrangement regions detected in the segmentation phase needs to be cleared of segments corresponding to non–pathogenic polymorphic changes, wave patterns, and spurious segments resulting from disrupted DNA probes.

### Our results

In our study, we have focused on *in silico* detection, and supervised verification, of rare CNVs (i.e. non–polymorphic and outstanding) underlying diverse spectrum of diseases in human. We analyze aCGH samples from a cohort of 366 patients (180 K probes custom exon–targeted CGH array [5]) from patients with developmental delay/intellectual disability, epilepsy, or autism. Patients were examined by Institute of Mother and Child, Warsaw, Poland (IMC). We develop and apply novel robust outliers detection procedure to identify aberration associated segments corresponding to the potentially pathogenic changes. We simultaneously process all accessible samples from patients to strengthen information about rearrangements patterns.

To this task we create a procedure which analyzes aCGH data from all samples (a logratio matrix), and detects short fragments of *k* consecutive probes (*k*–mers), which are markers of rare CNVs, and which are used to assign statistical, and clinical, significance to detected CNVs.

We augment typical normalizations steps with data transformation to ranks. We propose an outlier statistics, based on ranks, which identifies markers as lying in a 1% tail of the null distribution. This follows the definition of rare pathogenic CNVs, which are nearly absent in control population and present in 1% or less of affected individuals.

From the set of outstanding segments, we sieve out those corresponding to the non–pathogenic polymorphisms, and filter them basing on three main publicly available databases storing the information related to genomic variations and diseases: International Standards for Cytogenomic Arrays database (ISCA) [20], Genetic Association Database (GAD) [21] and Database of Genomic Variants (DGV) [22].

Our protocol results with a set of medically relevant CNVs. The validation sets the sensitivity of our method for rare CNVs detection to be 96%, and the specificity to be about 94%. We summarize the most interesting 18 CNV segments predicted by our method, that require further analysis (e.g. FISH) in the Validation section. These regions are suspected of being significant to autism, or to mental retardation.

A preliminary version of this paper was presented as extended abstract at IEEE International Conference on Bioinformatics and Biomedicine (BIBM 2011).

## Methods

### Datasets

The dataset comes from 366 arrays hybridized with DNA from subjects with epilepsy, autism, or other neurodevelopmental disorders (developmental delay/intellectual disability) examined at the IMC Cytogenetics Labs. Each experiment was performed on the 180 K custom whole–genome microarray with an exonic–coverage for over 1700 known and candidate genes for neurodevelopmental disorders [5].

Microarrays were prepared on Agilent platform, hybridized and scanned by Agilent scanner. We used Agilent Feature Extraction software with default settings, which performs back-ground subtraction, array spatial detrending, dye normalization and logratio calculations from each microarray [23,24].

For further analysis we used outputted logratios – each sample consists of a set of ∼180 K logratio intensities mapped to loci in the reference genome hg18 human assembly.

FISH, Multiplex ligation-dependent probe amplification (MLPA), or Polymerase chain reaction (PCR) methods were used for experimental validation.

### Overview

The overall architecture of our proposed method consists of three main phases: segmentation call, rare CNVs filtering, and segment significance testing.

For the segmentation phase, we decided to use DNACopy algorithm, based on recursively applying a statistical test to detect significant CNV breakpoints [25]. The algorithm, often proposed as a standard approach, processes aCGH data obtained from only a single experiment but is highly efficient and enables to analyze a new sample within a few minutes.

During the second phase, we apply the robust outliers detection procedure to identify aberration associated segment corresponding to the potentially pathogenic changes. In this stage, we simultaneously process all accessible samples to strengthen the information about the rearrangement patterns. We analyze the matrix of logratios, wherein the columns correspond to probes and rows to samples. The logratio signals are transformed into rankings in each column separately; this eliminates the influence of low quality probes and experimenter’s bias. Then, we calculate the distances between vectors from contiguous ranges of probes (called *k*-mers, i.e. windows of size *k*), based on the *L*_1_ (Manhattan) metrics. We model the distribution of the mean distances to all other *k*-mers in each window separately. We classify given *k*-mer as an outlier when it belongs to the distribution tail. Outliers correspond to markers of significantly outstanding DNA rearrangements and are mapped to the pre-existing segmentation. In the last step, we filter out polymorphic segments. If a specific threshold (based on density of coverage by markers and absolute value of segment’s mean logratio) is met, a segment along the patient genome is reported and selected to validation.

During the validation phase, following the methodology from Koolen, et al, we conduct exhausting comparison with three main databases storing information about genomic rearrangements and diseases: International Standards for Cytogenomic Arrays database (ISCA), Genetic Association Database (GAD) and Database of Genomic Variants (DGV) [7,20-22].

### Outstanding CNVs detection

Although logratio data is already normalized by microarray extraction software, we observe noisy patterns in it: wave bias and experimenter’s bias (Figure 1, also see Results and discussion). Wave bias has been documented in the literature before [19].

To overcome these two pertaining obstacles we propose an intuitive solution: the idea is to work with logratio signal relative to other samples, i.e. for any fixed probe to replace the logratios by their ranks in all samples. The highly beneficial effect of the algorithm is illustrated on Figure 1(a) and (b), which present the fragment of the genome with hybridization signal coded by logratios and their rankings, respectively. One can observe that both wave pattern (causing spurious segment calls) and disrupted probes are eliminated, while keeping the true positive segments (in this genome fragment one large deletion is visible).

Our procedure analyzes aCGH data from all samples (logratio matrix) to detect short fragments of *k* consecutive probes (*k*–mers) being the markers of rare CNVs.

The idea of markers is based on the definition of rare pathogenic CNVs, which are nearly absent in control population and present in 1% or less of affected individuals. Hence, we seek for markers in the set of *k*–mers for all samples (presented results were obtained for a parameter *k*=7). Outlier detection in high dimensional spaces is a non–trivial task. In our solution, we follow the recommendation from a survey of outlier detection methods by Gogoi, et al. to use a distance-based approach with a suitable choice of metrics [26].

We apply sliding window approach on a ranking transformed logratios matrix. For each window spanning the range of *k* columns, we calculate the distances between the *k*–mers from all samples. For each *k*–mer, we compare the average distance to all others in the same window. Then we approximate the distribution of average distances and classify the *k*–mer as a marker if it lies in a 1% tail of this distribution.

More formally, consider a logratio matrix *L* and one of its *k*-windows $L_{Q}^{S}$, containing logratio data coming from a set of patients S={1,…,n}, and from consecutive probes from the set *Q*={*p*,…,*p*+*k*−1} (here probe ordering respects probes positions on the reference genome). The transformation of each of *k* columns into ranks and division of resulting ranks by |*S*|+1 yields *pseudo–ranks* matrix $R_{Q}^{S}$ with elements:

$$R_{q}^{s}=\frac{\text{rank of}\;L_{q}^{s}\;\text{in}\;L_{q}^{S}}{|S|+1},\;s\in S,q\in Q$$

Notice, that each *k*-mer (row of $R_{Q}^{S}$) belongs to *k*-dimensional hypercube, i.e. $R_{Q}^{s}\in {\left(0,1\right)}^{k}$. The marginal distributions of pseudo-ranks for each one of *k* dimensions can be modeled as uniform^1^, while the join distribution of *k*-mers is not uniform on hypercube (0,1)^*k*^, because of correlated logratios from consecutive probes (e.g. the analyzed window covers a region of rearrangements).

Distributions with uniform marginals on hypercube (0,1)^*k*^ are commonly described using copulas [27]. *C* is a *k*-dimensional copula if it is a joint cumulative distribution function of a *k*-dimensional random vector on (0,1)^*k*^ with uniform marginals.

Our method for discriminating outliers is based on a statistics computed for each *k*-mer: *meanL*_*d*_*distance to other k-mers*. For *s*∈*S*, *d*∈(0, inf]:

$$\mu^{d}\left(s\right)=\frac{1}{\left|S\right|}\underset{j\in S}{\sum }{\left(\underset{l\in Q}{\sum }{\left|R_{l}^{s}-R_{l}^{j}\right|}^{d}\right)}^{\frac{1}{d}}$$

For Manhattan distance *d*=1 and in the simplified case of one dimension *k*=1 the value of the *μ* statistics for a patient with pseudo-rank *z*∈[ 0,1] converges with |*S*|→*∞* to:

$$\mu^{1}(z)=\int_{0}^{1}\;\left|t-z\right|\text{dt}=z^{2}+{\left(1-z\right)}^{2}$$

For *k*>1 if we undertake the independence of pseudo-ranked columns the null distribution $D_{\mu^{1}}^{k}$ of *μ*^1^ can be computed as a sum of independent variables. This underlines the adequacy of statistics *μ*^1^ as it converges to the sum of squared euclidean distances from two extreme corners of hypercube: 0^*k*^ and 1^*k*^ (a *k*-mer in each of the corners has extreme ranks on every probe, see Additional file 1 for details).

On the other hand, the null hypothesis may assume a certain structure of column correlations, e.g. corresponding to a larger group of patients with CNV segments inside a particular window, and a null distribution may reflect that. First approach we’ve taken is to fit as a null distribution Beta(*α*,*β*) shifted to the appropriate interval (min(*μ*^1^),max(*μ*^1^)). This outlier detection procedure is considered less conservative since Beta has a lighter tail than the $D_{\mu^{1}}^{k}$ for small *k* (see Additional file 1).

Second approach presupposes that the distribution of *k*-mers of pseudo-ranks is described by a certain copula *C*. Parameters of copula *C* are fitted for each window, the null distribution is obtained by integration of the *μ*^1^ statistics over copula *C* (see Additional file 1 for details). However, classical families of copulas (Gaussian, t-copula, Archimedean) are not suited to model multidimensional *k*-mers with asymmetric dimensional dependencies, a copulas mixture approach is more adequate [28]. Then, the mixture approach suffers from huge dimensionality – obtained solutions are only locally optimal, dependent on a mixture fitting starting point.

In either approach, *k*-mers with p-value less than 0.01 (suggested frequency of pathogenic CNVs) are selected as markers. Results presented in this paper originate from the first, Beta fit, approach.

Selected markers are lined up on the considered segmentation. We sieve out segments without any markers inside and sort segments that remain according to the density of coverage by markers (best scoring segments are most densely covered). We call the score assigned to reported segments *density score* in the sequel, as it corresponds to the percent of the segment covered by markers.

### Polymorphic regions filtering

Outlier statistic based method returns markers of rare CNVs, but also of some segments in highly polymorphic regions, where background distribution strongly diverges from the null distribution. Thus, from the set of outstanding segments covered by markers, we sieve out those corresponding to the non–pathogenic polymorphisms. To this aim we construct a so called polymorphic profile as follows.

The segmentation is mapped into probes, i.e. to each probe we assign the value of the mean logratio in the segment containing it. The signal is considered as not–noisy if its absolute value exceeds 0.07. This corresponds to log2ratio=0.24 which is a conservative (high) threshold for aberration detection. We count the number of signals above this threshold in each column *i* and if there are more than three (1% of 366 samples) we set the *i*-th coordinate in the polymorphic profile to 1, otherwise it is set to 0.

Next, we scan the profile vector and identify all *k*-mers (*k*=7) of consecutive ones. At the end, the set of outlier *k*-mers from the previous step is intersected with the full set of *k*-mers, that indicates the polymorphism. Outlier *k*-mers that overlap with polymorphic profile are excluded from further analysis.

### Validation

To validate segments selected as rare CNVs according to our density score we automate the process of a manual validation of segments based on UCSC [29], i.e. the protocol by which geneticians usually act. Lastly, we compare resulting sets of segments with the set produced manually by geneticists from IMC.

Manual validation by geneticists involves inspecting reported CNVs segments, overlaying them on UCSC tracks. This purposes to filter out known polymorphisms and, by interrogation of all known syndrome regions, to try to narrow down the segment set to only those clinically relevant. This step is followed by FISH or PCR confirmation of the CNVs existence in patient’s DNA [30,31].

For the automated process we decided to focus on three main databases storing the information related to genomic variations and diseases resulting from it: ISCA, DGV and GAD.

ISCA is a group of clinical cytogenetics laboratories committed to improve the quality of patients care related to clinical genetic testing using aCGH [20]. ISCA database contain very high quality copy number data (i.e. deletions and duplications) from clinical laboratories. Currently, ISCA stores 1.7 Gb of CNVs data classified as pathogenic.

The objective of the DGV is to provide a comprehensive summary of structural variation in the human genome identified in healthy control samples [22]. Currently DGV is used to justify the rarity of identified rearrangement. However, this approach is criticized because DGV contain many false positives (e.g. small aberrations detected by poor–resolution technology are considered to be larger) [7]. On the other hand, its content is not representative for analysis of specific group of patients. Therefore, our procedure for polymorphisms filtering is based on in house database, as suggested [7].

The GAD is an archive of human genetic association studies of complex diseases and disorders allowing for rapid identification of medically relevant polymorphisms from the large volume of polymorphism data [21].

In our validation approach, we investigate the correlation of marker coverage density score for segments with the contents of described databases. For medically relevant CNVs, we expect insignificant intersection with DGV and a non–empty intersection with ISCA and/or GAD.

### Ethical approval

Informed consents approved by the Institutional Review Board of the Bioethics Committee at the IMC (12/2007) were obtained in all cases.

## Results and discussion

### Adequacy of density score

**Figure 2:**
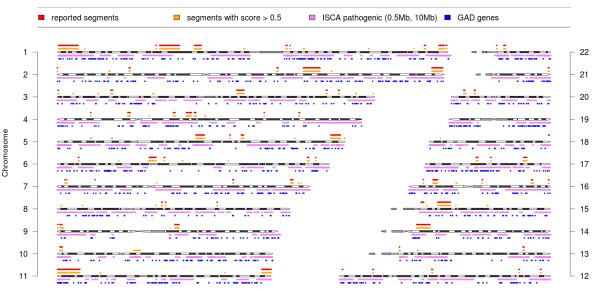
**Rare CNVs detected by our method in 366 samples.** Figure shows the chromosmal location of all segments reported by experts (red), segments predicted by our method (yellow) as well as pathogenic CNVs reported in ISCA (purple) and genes from GAD (blue).

A central challenge in CNV–disease association studies is to characterize the pathogenicity of rare CNV events [7]. It is usually done by a manual inspection of several UCSC tracks [29]. In our approach, we have implemented this process as follows. For each predicted segment (achieving predefined level of density score), we calculate the number of genomic variants in healthy individuals (according to DGV), which have a significant intersection with our segment. Moreover, we scan the GAD database for genetic associations and ISCA database for pathogenic events located in this region, see Figure 2.

**Figure 3:**
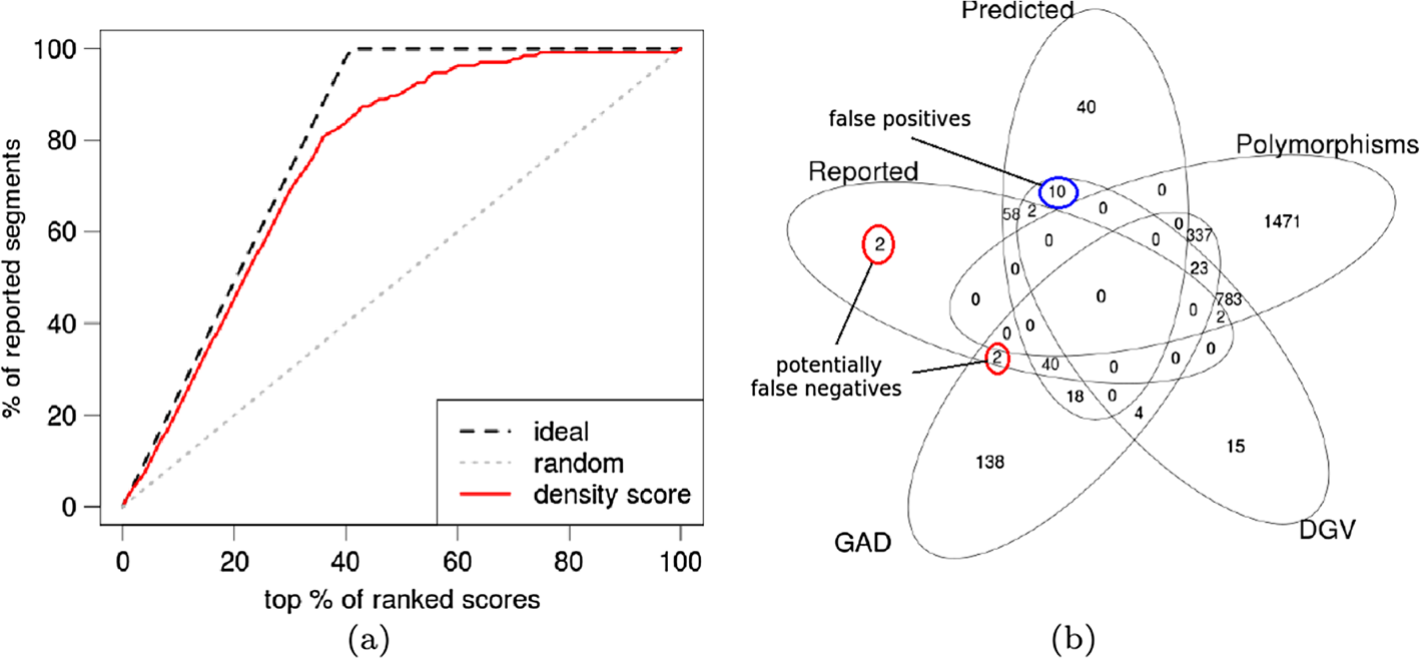
**Evaluation of CNVs detection results.(a)** The enrichment curve for a density score with respect to the segments already reported as pathogenic or of uncertain significance. **(b)** Venn diagram for the predicted rare CNVs (Predicted), confirmed as pathogenic or uncertain (Reported), segments significantly overlapping with DGV (DGV), segments with GAD genes (GAD), and segments selected as polymorphisms according to polymorphic profile (Polymorphisms).

To illustrate the adequacy of our density score, we calculated the enrichment curve with respect to segments already confirmed as pathogenic or reported as those of uncertain significance that needs further analysis (see Figure 3(a)). The diagonal depicts the enrichment curve in the case of a random selection of segments, the dashed line is the ideal enrichment curve and the red line is the enrichment curve for our density score.

Figure 3(b) presents the Venn diagram for five subsets of the set of ca. 3000 segments having absolute mean logratio value greater than 0.1. Inside this set we consider: set of all reported segments, set of segments predicted by us (having density score above 50%), set of segments containing GAD genes, set of segments with significant intersection with DGV (at least 3 cases) and the set of segments classified as polymorphic according to our filtering procedure based on polymorphic profile. Observe that the filtering method eliminated 98% of segments with DGV content, and more importantly 1808 other segments, which are also polymorphic but not reported in DGV. The sensitivity of our method for rare CNVs detection is 96% (only 4 segments reported by experts are missed from all 102 reported but non-DGV segments) and the specificity is about 94% (as false positives we classify 10 predicted but non-reported segments having significant DGV intersection). The most interesting findings that require further analysis (e.g. FISH) are 18 segments predicted by our method containing GAD regions.

### Robust analysis via rankings

The wave bias, as depicted on Figure 1, seems to be platform–independent and occurs in any high–resolution DNA hybridization studies. The usefulness of the wave smoothing methods proposed thus far can be more prob lematic than beneficial, e.g. method proposed by van de Wiel, *et al* needs the calibration profiles containing clear wave patterns and is applicable only to tumor data (not genomic disorders) [19]. Therefore, some authors prefer to simply remove samples with extreme wave factors from further analysis then to risk the loss of a significant segment.

Another very important but still neglected issue concerns the burden caused by a specific technician, so called experimenter’s bias. In one of our preliminary studies, for the considered cohort of patients, we obtained perfect discrimination (based on only few disrupted probes) between groups of samples analyzed by a specific technician (data not shown).

### Discovery and validation of rare CNVs

In a cohort of 366 patients, our method identified 168 potentially pathogenic duplications and deletions that met coverage density score 50%. From this set, 100 changes have been reported (i.e. already confirmed (by FISH) as real pathogenic and non–pathogenic changes or selected as changes of unknown significance for further analysis). Most pathogenic CNVs correspond to deletions, as there are many fewer pathogenic events associated with amplifications.

**Figure 4:**
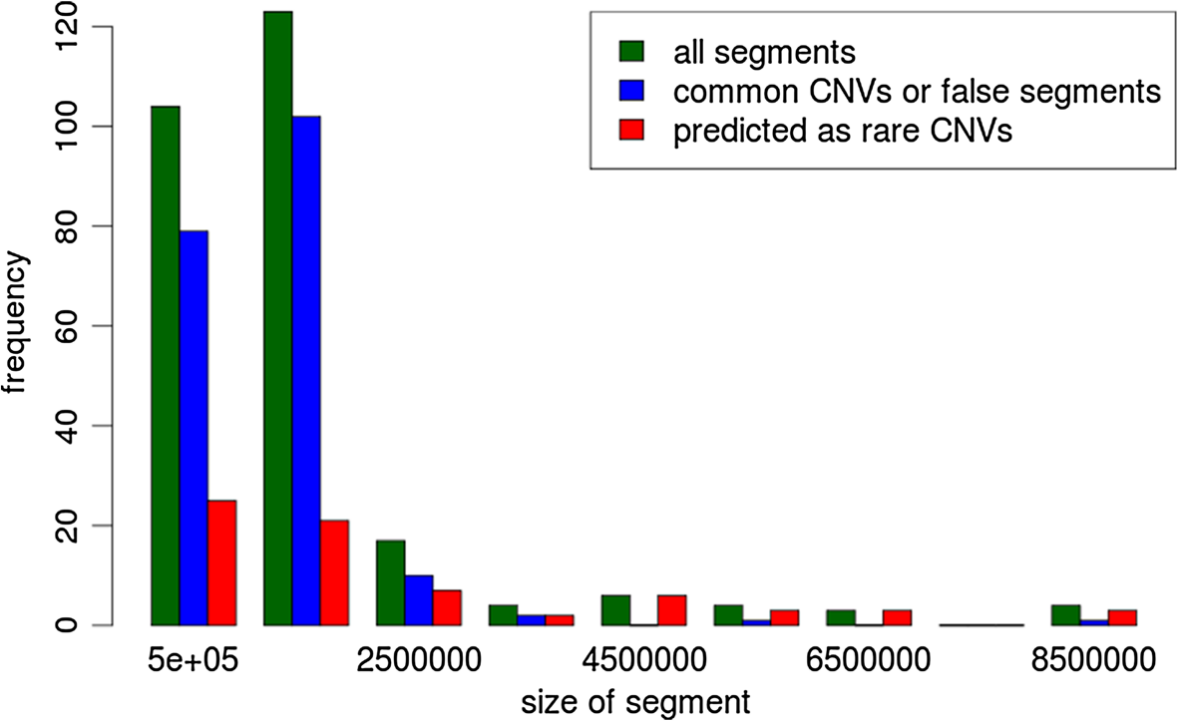
**The distribution of lengths of detected CNVs.** Green bars correspond to the distribution of lengths of all detected segments. Blue bars indicate to the frequencies of common CNVs or false positive segments, while red bars represent segments predicted as rare CNVs.

Table 1 presents selected best scoring CNVs predicted by us and confirmed (by karotype or FISH) to be pathogenic, while Table 2 lists predicted best scoring CNVs of uknown significance, which are currently being investigated using FISH or PCR. Figure 4 shows the distribution of segments’ lengths: rare CNVs predicted by us are much longer than common CNVs or false positive segments, which is consistent with previous analysis [32].

**Table 1:** Selected predicted best scored CNVs confirmed later as pathogenic changes

**Case**	**Gain/loss**	**Cytoband**	**Size (Mb)**	**Oligo nr.**	**Score**	**ISCA**	**GAD**	**Diagnosis**
1	Del	1q43q44	3.3	166	100%	24	—	Mental retardation
2	Del	3q13.2q13.31	4.5	154	99%	4	2	Autism
3	Del	Xp22.12	1.6	100	95%	53	1	Mental retardation
4	Del	17q21.31	0.3	84	96%	23	3	Mental retardation
5	Del	5q14.3q15	5.4	596	95%	5	—	Mental retardation
6	Del	Xq22.1q22.3	5.2	167	91%	51	2	Mental retardation
7	Del	2q37.2q37.3	6.3	736	88%	19	2	Mental retardation
8	Del	15q13.3q14	8	873	87%	2	2	Mental retardation

**Table 2:** Selected predicted best scored variants of unkown significance

**Case**	**Gain/loss**	**Cytoband**	**Size (Mb)**	**Oligo nr.**	**Score**	**ISCA**	**Diagnosis**
1	Del	8q22.2	0.25	56	100%	5	Autism
2	Del	5q35.3	0.7	27	93%	6	Mental retardation
3	Dup	3p26.3	0.33	21	90%	10	Mental retardation
4	Dup	12q24.32	0.4	9	88%	7	Mental retardation
5	Dup	4q28.2	0.12	77	87%	6	Mental retardation
6	Dup	3p22.3	1.2	30	83%	4	Autism
7	Dup	6q25.3	0.9	17	82%	4	Mental retardation
8	Del	4q21.23q21.3	0.95	22	81%	4	Autism

## Conclusions

Many recent studies have emphasized the role of CNVs in the etiology of many human diseases, with rare variants being particularly important [33]. Current methods for detection of CNVs in individual samples are not capable to infer such information, while most approaches for multi sample analysis focus on frequent CNVs present in tumor samples. We propose the efficient solution filling this gap that can be used for accurate detection of rare CNVs and has potential use in clinical diagnostics. Since our procedure produces a set of markers for rare CNVs, it may be efficiently used to filter a segmentation produced by any other segmentation algorithm, and help with identification of segments corresponding to rare pathogenic polymorphisms.

The ongoing study on a group of 366 individuals confirmed large part of our predictions (see previous section, Tables 1 and 2).

Moreover, the validation of the proposed segments scoring indicates the significant enrichment of high scoring segments in disease genes from GAD database and impoverishment in benign CNVs present in DGV database. Futhermore, the extensive intersection of rearrangements detected by us with data stored in ISCA indicates the potential pathogenic changes in our segments.

The presented method is robust in the sense of sensitivity to outliers coming from spurious probes, or any singular outliers of other type, when comparing to segmentation on each sample separately (DNACopy algorithm was used in this study). Last but not least, it is also resistant to waviness. The DNA–copy segmentation algorithm used in the first stage of our method can be replaced by any other procedure, and more importantly it can be also skipped at all. In that case, we can cluster the significance markers found during the second phase along the genome to obtain longer segments. This idea leads to multi-sample segmentation algorithm that can be highly efficient and we plan to exploit it in the future.

## Competing interests

The authors declare that they have no competing interests.

## Authors’ contributions

MS, TG and AG developed algorithms used for CNVs detection. MB, KD, BW-K, and PS performed aCGH experiments and provided the detailed description of the results. MS, AG and TG wrote the manuscript. All authors reviewed and approved of the final manuscript.

## Supplementary Material

Additional file 1 **Supplementary information.** This is an extended section Methods/Outstanding CNVs detection from Sykulski et al. “Multiple samples aCGH analysis for rare CNVs detection”, where the statistics mean *L*_*q*_ distance to other rank vectors is analyzed in greater detail. The statistics is used to select outlier rows from logratio data matrix resulting from stacking ACGH (Array Comparative Genomic Hybridization) results from many patients. The robust statistical framework applied in our method enables to eliminate the in uence of widespread technical artifact termed ‘waves’. Click here for file
