# Supplementary material for: Multiple samples aCGH analysis for rare CNVs detection
Source: J Clin Bioinforma. 2013 Jun 11;3:12. doi: 10.1186/2043-9113-3-12 (PMC3691624; doi:10.1186/2043-9113-3-12)
Supplement: Additional file 1 — Supplementary information. This is an extended section Methods/Outstanding CNVs detection from Sykulski et al. “Multiple samples aCGH analysis for rare CNVs detection”, where the statistics mean Lq distance to other rank vectors is analyzed in greater detail. The statistics is used to select outlier rows from logratio data matrix resulting from stacking ACGH (Array Comparative Genomic Hybridization) results from many patients. The robust statistical framework applied in our method enables to eliminate the in uence of widespread technical artifact termed ‘waves’. [file 2043-9113-3-12-S1.pdf]

Supplementary Information - Multiple samples aCGH analysis for rare CNVs detection

Maciej Sykulski[1], Tomasz Gambin[2], Magdalena Bartnik[3], Katarzyna Derwińska[3], Barbara Wiśniowiecka-Kowalnik[3], Paweł Stankiewicz[3,4] and Anna Gambin*[1,5]

[1]Institute of Informatics, University of Warsaw, Warsaw, Poland
[2]Institute of Computer Science, Warsaw University of Technology, Warsaw, Poland
[3]Dept of Medical Genetics, Institute of Mother and Child, Warsaw, Poland
[4]Dept of Molecular and Human Genetics, Baylor College of Medicine, Houston, TX, USA
[5]Mossakowski Medical Research Centre, Polish Academy of Sciences, Warsaw, Poland

Email: Maciej Sykulski - macieksk@mimuw.edu.pl; Tomasz Gambin - tgambin@ii.pw.edu.pl; Magdalena Bartnik - magdalena.bartnik@imid.med.pl; Katarzyna Derwińska - katarzyna.derwinska@imid.med.pl; Barbara Wiśniowiecka-Kowalnik - barbara-wisniowiecka@tlen.pl; Paweł Stankiewicz - pawels@bcm.edu; Anna Gambin*- aniag@mimuw.edu.pl;

*Corresponding author

Abstract

This is an extended section *Methods/Outstanding CNVs detection* from Sykulski et al. "Multiple samples aCGH analysis for rare CNVs detection", where the statistics *mean L_q distance to other rank vectors* is analyzed in greater detail. The statistics is used to select outlier rows from logratio data matrix resulting from stacking ACGH (Array Comparative Genomic Hybridization) results from many patients.

The robust statistical framework applied in our method enables to eliminate the influence of widespread technical artifact termed 'waves'.

Methods

Outstanding CNVs detection

Although logratio data is already normalized by microarray extraction software, we observe noisy patterns in it: wave bias and experimenter's bias (Figure 1, also see Discussion). Wave bias has been documented in the literature before [1].

To overcome these two pertaining obstacles we propose an intuitive solution: the idea is to work with

logratio signal relative to other samples, i.e. for any fixed probe to replace the logratios by their ranks in all samples. The highly beneficial effect of the algorithm is illustrated on Figure 1 (a) and (b), which present the fragment of the genome with hybridization signal coded by logratios and their rankings, respectively. One can observe that both wave pattern (causing spurious segment calls) and disrupted probes are eliminated, while keeping the true positive segments (in this genome fragment one large deletion is visible).

Our procedure analyzes aCGH data from all samples (logratio matrix) to detect short fragments of k consecutive probes (k–mers) being the markers of rare CNVs. The idea of markers is based on the definition of rare pathogenic CNVs, which are nearly absent in control population and present in 1% or less of affected individuals. Hence, we seek for markers in the set of k–mers for all samples (presented results were obtained for a parameter $k = 7$). Outlier detection in high dimensional spaces is a non–trivial task. In our solution, we follow the recommendation from a survey of outlier detection methods by Gogoi, et al. to use a distance-based approach with a suitable choice of metrics [2].

We apply sliding window approach on a ranking transformed logratios matrix. For each window spanning the range of k columns, we calculate the distances between the k–mers from all samples. For each k–mer, we compare the average distance to all others in the same window. Then we approximate the distribution of average distances and classify the k–mer as a marker if it lies in a 1% tail of this distribution.

More formally, consider a $\log_2$ratio matrix L and one of its k-windows L_Q^S, containing $\log_2$ratio data coming from a set of patients $S = \{1, \ldots, n\}$, and from consecutive probes from the set $Q = \{p, \ldots, p+k-1\}$ (here probe ordering respects probes positions on the reference genome). The transformation of each of k columns into ranks and division of resulting ranks by $|S| + 1$ yields *pseudo–ranks* matrix R_Q^S with elements:

$$R_q^s = \frac{\text{rank of } L_q^s \text{ in } L_q^S}{|S| + 1}, \quad s \in S, q \in Q$$

Let us consider, that S is a patient group sampled from a large group of all patients $\mathcal{S}$, and that rows of R^S contained in $[0, 1]^k$, are in fact pseudo-ranks in columns of $\mathcal{S}$, respectively. Now, R_q^s, taken from a random patient s and probe q, has uniform distribution. Hence, R_Q^S is a sample from distribution $\mathcal{D}_p$ with *c.d.f.* $D_p : [0, 1]^k \to [0, 1]$ with uniform marginals: $D_p (1, \ldots, u_i, \ldots, 1) = u_i \quad \forall i$. However, observe, that if one or more patients in the sample exhibit CNV segment, columns $R_{q \in Q}^S$ are correlated with each other, hence $\mathcal{D}_p$ is not uniform on $[0, 1]^k$. In statistics, distributions with uniform marginals on a hyper-cube $[0, 1]^k$ are commonly described using copulas. C is a k-dimensional copula if C is a joint cumulative distribution function of a k-dimensional random vector on the unit cube $[0, 1]^k$ with uniform marginals. Several families of copulas (Gaussian copulas, t-copulas, Archimedean copulas), and their properties, were thoroughly studied

in literature.

Our method for discriminating outliers is based on a statistics computed for each of n patients: *mean L_q distance to other rank vectors.*

$$\mu^q(s) = \frac{1}{|S|} \sum_{j \in S} \left(\sum_{l=p}^{p+k-1} \left| R_l^s - R_l^j \right|^q \right)^{\frac{1}{q}} \quad , \quad s \in S,\ q \in (0, \inf]$$

For the purpose of this work we selected L_1 distance measure, both for simplicity and greater robustness than L_2.

In the case of one dimension $k = 1$ and in the continuous limit $|S| \to \inf$, the value of the μ^1 statistics for a patient with pseudo-rank $z \in [0, 1]$ is given by:

$$\mu^1(z) = \int_0^1 |t - z|\, dt = z^2 + (1 - z)^2$$

$\mu^1(z)$ is monotonous over $z \in [0, \frac{1}{2}]$, and symmetric with respect to $\frac{1}{2}$, z has uniform distribution. Substituting $u = 2|z - \frac{1}{2}|$ we obtain the inverse cumulative distribution function, and further the cdf and the density of the null distribution for $k = 1$.

$$\mathrm{F}_{\mu^1}^{-1}(u) = \left(\frac{1+u}{2}\right)^2 + \left(\frac{1-u}{2}\right)^2 = \frac{u^2+1}{2} \quad , \quad u \in [0, 1]$$

$$\mathrm{F}_{\mu^1}(x) = \sqrt{2x - 1} \quad , \quad g_{\mu^1}(x) = \frac{1}{\sqrt{2x-1}} \quad , \quad x \in [\frac{1}{2}, 1]$$

For $k > 1$ the value of the μ^1 statistics for a patient with pseudo-ranks $z = (z_1, \ldots, z_k) \in [0, 1]^k$ is given by:

$$\mu^1(z) = \sum_{i=1}^{k} \int_0^1 |t - z_i|\, dt = \sum_{i=1}^{k} z_i^2 + \sum_{i=1}^{k} (1 - z_i)^2 = ||z||_2^2 + ||1^k - z||_2^2$$

This signifies that the μ^1 statistics converges in limit $|S| \to \infty$ to the sum of squared euclidean distances from two extreme corners of hypercube: 0^k and 1^k (a k-mer in each of these corners has extreme ranks on every probe).

For $k > 1$ if we undertake the independence of pseudo-ranked columns the null distribution $D_{\mu^1}^k$ of μ^1 can be computed as a sum of independent variables. This underlines the adequacy of statistics μ^1 as it converges to the sum of squared euclidean distances from two extreme corners of hypercube: 0^k and 1^k (a k-mer in each of the corners has extreme ranks on every probe). Figure 2 presents μ^1 limit $|S| \to \infty$ null distributions for various dimensions k for the dimension independence case.

On the other hand, the null hypothesis may assume a certain structure of column correlations, e.g. corresponding to a larger group of patients with CNV segments inside a particular window, and a null distribution

may reflect that. First approach we've taken is to fit as a null distribution $\mathrm{Beta}(\alpha, \beta)$ shifted to the appropriate interval $(\min(\mu^1), \max(\mu^1))$. This outlier detection procedure is considered less conservative since Beta has a lighter tail than the $D^k_{\mu^1}$ for small k.

Second approach presupposes that the distribution of k-mers of pseudo-ranks is described by a certain copula C. In case the rank distribution is a certain copula $\mathcal{D}_p = C$, the *c.d.f.* of the null distribution $\mathcal{F}_\mu$ is estimated through approximation of the following integral, by either computing it numerically, or through sampling from the fitted copula C:

$$\mathcal{F}_\mu(m) = \int_{[0,1]^k} \mathbb{1}_{\mathcal{F}_\mu^{-1}(z_1,\ldots,z_k) \leq m} d\mathcal{D}_p(z_1, \ldots, z_k) = \int_{[0,1]^k} \mathbb{1}_{\sum_{i=1}^k F_\mu^{-1}(z_i) \leq m} dC(z_1, \ldots, z_k)$$

Parameters of copula C are fitted for each window, the null distribution is obtained by integration of the μ^1 statistics over copula C. However, classical families of copulas (Gaussian, t-copula, Archimedean) are not suited to model multidimensional k-mers with asymmetric dimensional dependencies, a copulas mixture approach is more adequate [3]. Then, the mixture approach suffers from huge dimensionality – obtained solutions are only locally optimal, dependent on a mixture fitting starting point.

In either approach, k-mers with p-value less than 0.01 (suggested frequency of pathogenic CNVs) are selected as markers. Results presented in this paper originate from the first, Beta fit, approach.

Selected markers are lined up on the considered segmentation. We sieve out segments without any markers inside and sort segments that remain according to the density of coverage by markers (best scoring segments are most densely covered). We call the score assigned to reported segments *density score* in the sequel, as it corresponds to the percent of the segment covered by markers.

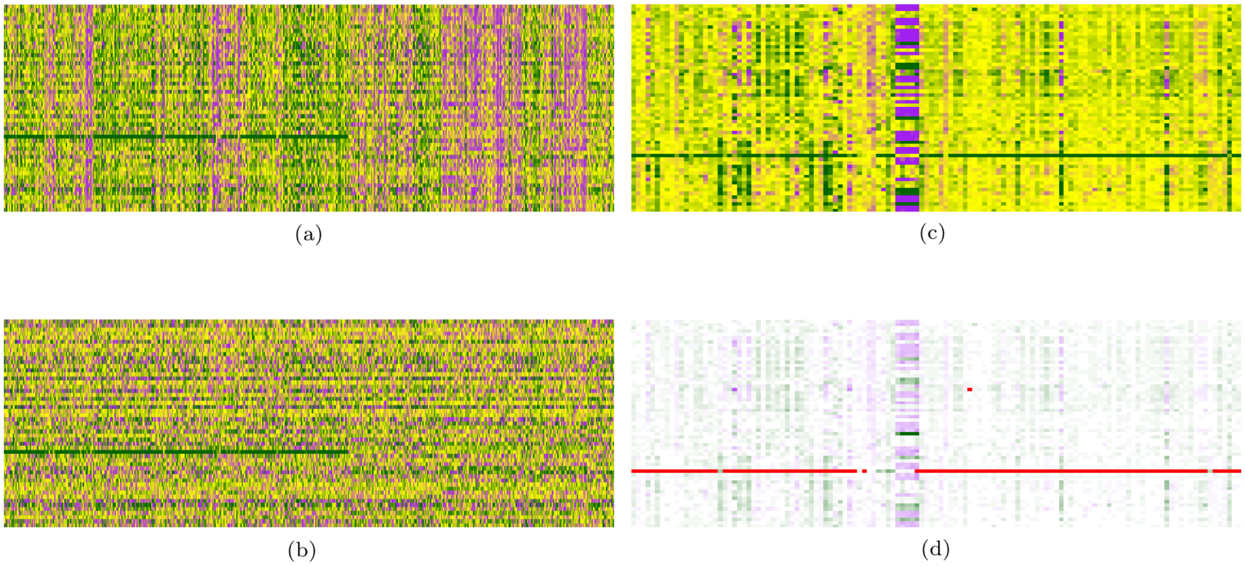

Figure 1: **Processing of logratio data/** In each subfigure, rows corresponds to samples and columns to probes. On the left: the effect of rank transformation; the same fragment of the genome represented by logratios (a) and their column ranks (b). The wave pattern is eliminated, while true signal (clear deletion) is strengthen. On the right: the polymorphic region in the middle is surrounded by wave patterns and only one significant deletion is visible (c); markers found by our algorithm indicate only deleted segment, all other spurious signals are ignored (d).

References

1. van de Wiel MA, et al.: **Smoothing waves in array CGH tumor profiles**. *Bioinformatics* 2009, **25**(9):1099–1104.

2. Gogoi P, et al.: **A Survey of Outlier Detection Methods in Network Anomaly Identification**. *The Computer Journal* 2011, **54**:570–588.

3. Tewari A, Giering MJ, Raghunathan A: **Parametric Characterization of Multimodal Distributions with Non-gaussian Modes**. In *2012 IEEE 12th International Conference on Data Mining Workshops, Volume 0*, Los Alamitos, CA, USA: IEEE Computer Society 2011:286–292.

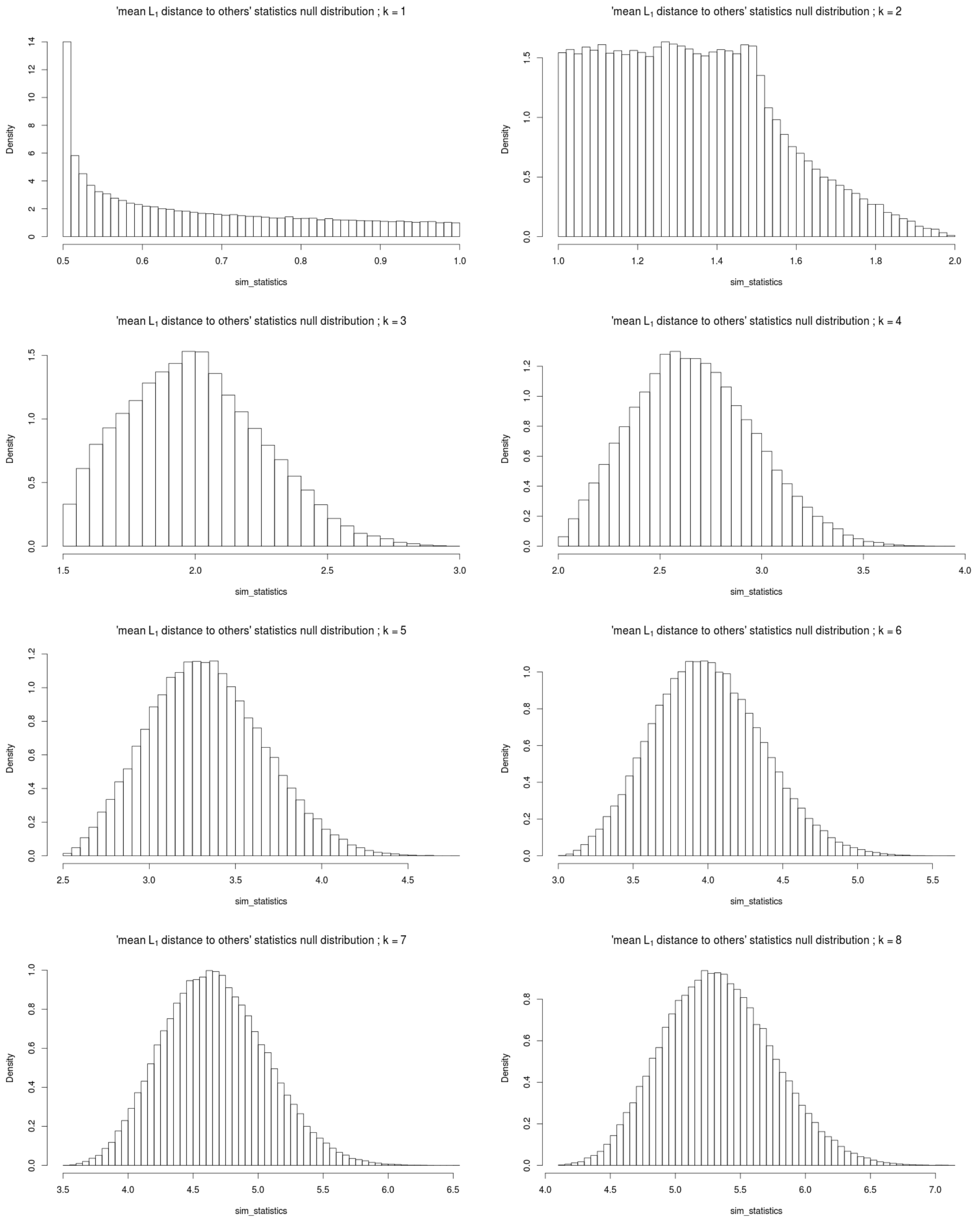

Figure 2: This figure presents histograms from samples from μ^1 (L_1 distance) null distributions (limit $|S| \to \infty$, number of cases converging to infinity) for various dimensions k. This sampling undertakes the assumption of column (dimensions) independence.
